# Clinical Reference Values for Dynamic Plantar Pressure Assessment in Healthy Young Adults: Sex Differences and Bilateral Symmetry

**DOI:** 10.3390/life16091449

**Published:** 2026-08-31

**Authors:** Daniela Matei, Elena Taina Avramescu, Ana Maria Bumbea, Simona Pătru, Dănuț Visarion Caimac, Anca Maria Amzolini, Carmen Daniela Neagoe, Gheorghe Ionescu, Klejda Tani, Miruna Andreiana Matei, Sergiu Cazacu, Diana Clenciu, Adina Mitrea, Mihai Cealîcu, Magdalena Rodica Trăistaru

**Affiliations:** 1 Department of Medical Rehabilitation, University of Medicine and Pharmacy of Craiova, 200349 Craiova, Romania; daniela.matei@umfcv.ro (D.M.); anmaria.bumbea@umfcv.ro (A.M.B.); simona.patru@umfcv.ro (S.P.); danut.caimac@umfcv.ro (D.V.C.); 2 Department of Sports Medicine and Kinesiology, University of Craiova, 200585 Craiova, Romania; taina_mistico@yahoo.com (E.T.A.); gheorghe.ionescu@edu.ucv.ro (G.I.); rodica.traistaru@umfcv.ro (M.R.T.); 3 Department of Medical Semiology, University of Medicine and Pharmacy of Craiova, 200349 Craiova, Romania; 4 Department of Internal Medicine, University of Medicine and Pharmacy of Craiova, 200349 Craiova, Romania; daniela.neagoe@umfcv.ro; 5 Department of Rehabilitation, Sport University of Tirana, 1001 Tirana, Albania; ktani@ust.edu.al; 6 Faculty of Medicine, University of Valencia, 46010 Valencia, Spain; andreian@alumni.uv.es; 7 Department of Gastroenterology, University of Medicine and Pharmacy of Craiova, 200349 Craiova, Romania; sergiu.cazacu@umfcv.ro; 8 Department of Diabetes, Nutrition and Metabolic Diseases, University of Medicine and Pharmacy of Craiova, 200349 Craiova, Romania; diana.clenciu@umfcv.ro (D.C.); adina.mitrea@umfcv.ro (A.M.); 9 Faculty of Medicine, University of Medicine and Pharmacy of Craiova, 200349 Craiova, Romania; mihaicealicu18@gmail.com

**Keywords:** dynamic plantar pressure, clinical reference values, gait analysis, bilateral symmetry, sex differences, pedobarography

## Abstract

This study aimed to establish a sex- and side-specific, platform- and population-specific descriptive reference dataset for dynamic plantar loading in healthy young adults and to investigate regional variability, mean left–right differences, and differences between female and male participants. Methods: A cross-sectional observational study included 90 healthy young adults selected from 102 initially recruited participants after predefined quality-control procedures. Bilateral plantar loading recordings were obtained using the RSscan platform under standardized conditions. Maximum force, impulse, and contact area were analyzed as the primary reference parameters across ten anatomical plantar regions, while load rate was additionally evaluated in the variability analysis. Sex- and side-specific descriptive statistics were calculated, and inter-individual variability was assessed using the coefficient of variation. Mean left–right differences were evaluated using paired Student’s *t*-tests. Differences between female and male participants were evaluated separately for each side using Welch’s independent-samples *t*-tests, with Benjamini–Hochberg false discovery rate correction for multiple comparisons. Results: Sex- and side-specific descriptive reference values were obtained for maximum force, impulse, and contact area. Maximum force and impulse were highest in the heel and central metatarsal regions, whereas contact area was greatest in the midfoot and heel. Contact area showed comparatively low inter-individual variability across plantar regions, whereas maximum force exhibited greater regional variability, particularly in the forefoot. Significant differences between female and male participants were observed for several parameters and plantar regions after correction for multiple comparisons. No statistically significant mean side differences were detected between corresponding plantar loading parameters of the left and right feet (all *p* > 0.05); this finding should not be interpreted as evidence of individual bilateral symmetry or equivalence. Conclusions: The present study provides a platform- and population-specific descriptive reference dataset for plantar loading in healthy young adults assessed using the RSscan system under standardized acquisition conditions. The reported values may serve as descriptive benchmarks for assessments performed using comparable measurement conditions; however, they should not be interpreted as diagnostic thresholds. Further validation in larger and more diverse populations and across different plantar pressure measurement systems is required before broader clinical application.

## 1. Introduction

Walking, although seemingly simple, represents a complex biomechanical process involving coordinated interactions between joints, muscles, and neuromuscular control mechanisms [1]. While general gait patterns are shared across individuals, subtle inter-individual variations are always present, contributing to the uniqueness of each walking pattern [2,3].

Gait analysis is widely used in clinical practice and sports biomechanics for the objective evaluation of movement patterns [4], while recent technological advances have further improved the detection of subtle biomechanical abnormalities, even in asymptomatic individuals.

Although experienced clinicians can derive valuable information through visual observation, the complexity and speed of human movement limit the accuracy of subjective assessment [5,6]. Therefore, the use of instrumental techniques has become essential for obtaining objective, quantitative, and reproducible data.

Modern gait analysis integrates motion analysis, ground reaction force measurements, and electromyography, providing a comprehensive understanding of locomotor function [7]. Among these techniques, dynamic plantar pressure assessment offers detailed insights into foot–ground interaction, including regional force distribution, plantar contact patterns, and loading characteristics during gait [8].

However, despite its widespread use, the interpretation of plantar pressure data remains challenging. One of the main limitations is the lack of standardized reference values applicable across different populations and plantar pressure measurement systems [9,10,11,12,13,14]. Previous studies have demonstrated considerable variability in plantar pressure parameters, influenced by subject characteristics, measurement protocols, and device-specific factors [10,11]. Systematic reviews and meta-analyses have further shown that differences in study populations, acquisition protocols, and measurement systems limit the comparability of plantar pressure data and hinder the establishment of broadly applicable reference values [12,13,14].

Additionally, plantar pressure distribution is influenced by multiple factors, including sex, anthropometric characteristics, and walking conditions. Previous studies have shown that sex-related differences exist in plantar loading patterns, affecting both force distribution and contact characteristics [13]. In addition, variability in plantar pressure measurements has been reported even in healthy populations, emphasizing the importance of standardized acquisition protocols and cautious interpretation of normative data [9,10].

Another important aspect is bilateral symmetry. Although normal gait is generally considered symmetrical, minor asymmetries may occur and are not necessarily indicative of pathology. Instead, they may reflect functional adaptations or natural biological variability [15,16].

In this context, the establishment of reliable platform- and population-specific reference values in homogeneous populations may improve the clinical interpretation of dynamic plantar pressure assessment. Such reference datasets may facilitate the interpretation of plantar loading patterns during functional evaluation and provide descriptive normative benchmarks for comparison with pathological populations presenting altered gait mechanics.

We hypothesized that dynamic plantar loading in healthy young adults would show consistent regional loading patterns, no systematic mean left–right differences, and measurable sex-related differences in selected biomechanical parameters. Furthermore, we expected that certain plantar regions would exhibit lower inter-individual variability, thereby providing more consistent descriptive reference data for functional assessment and future research applications.

This study set out to determine whether dynamic plantar loading in healthy young adults shows consistent regional patterns, whether systematic mean left–right differences are present, and whether measurable sex-related differences can be identified, with the goal of establishing clinically applicable, platform-specific descriptive reference values.

## 2. Materials and Methods

### 2.1. Study Design and Participants

This cross-sectional observational study was conducted with the aim of establishing clinically applicable sex- and side-specific reference values for dynamic plantar pressure assessment in healthy young adults using a standardized pedobarographic protocol. A total of 102 physically active young adults without known lower-limb pathology or gait abnormalities were initially recruited from the Faculty of Physical Education and Sports. All participants underwent bilateral dynamic plantar pressure assessment using the RSscan pressure platform under standardized testing conditions.

**Figure 1:**
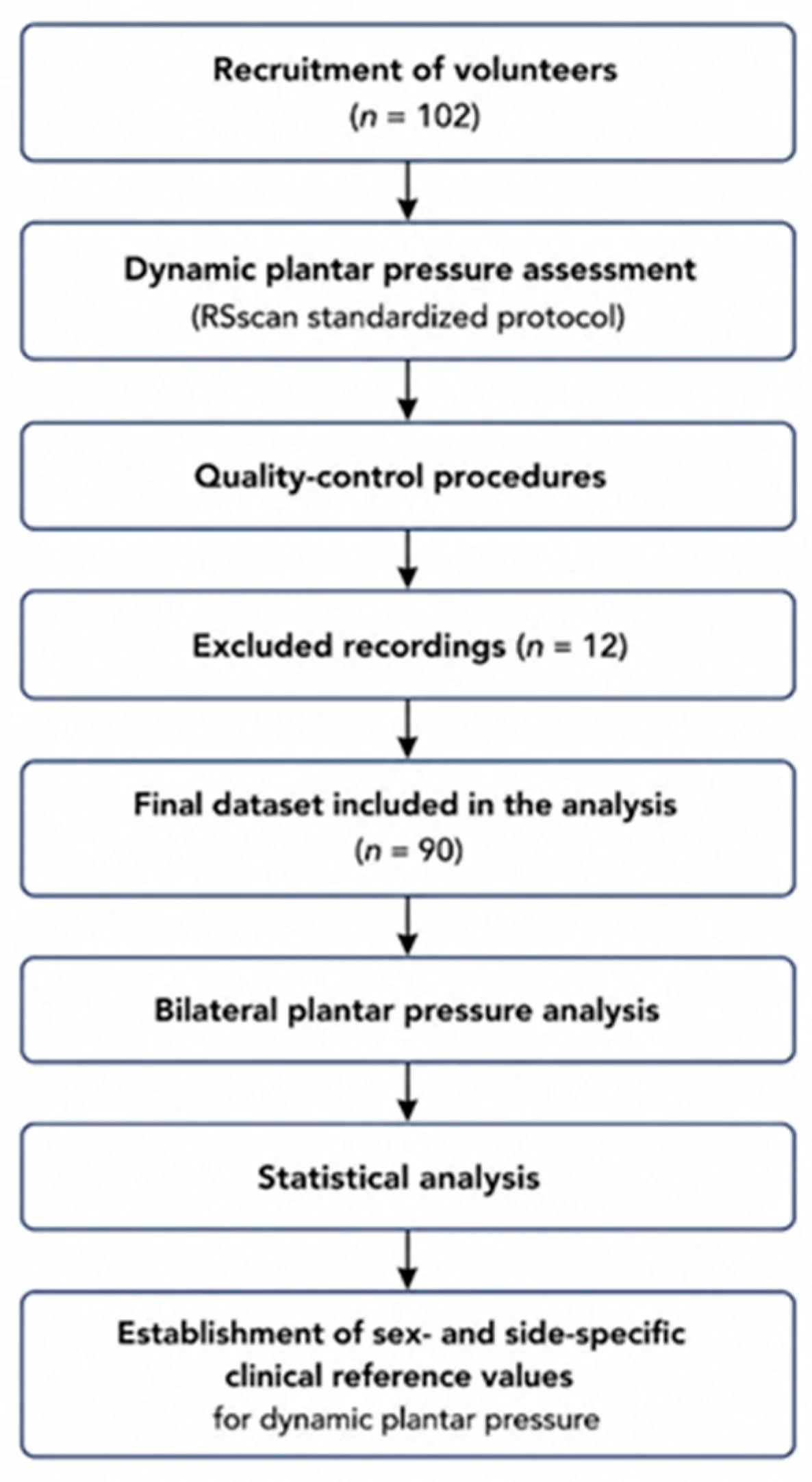
Study workflow. Flow diagram illustrating participant recruitment, dynamic plantar pressure assessment, data-screening procedures, statistical analysis, and generation of the sex- and side-specific platform-specific reference dataset.

Following data screening, 12 participants (7 females and 5 males) were excluded because one or more of their recorded biomechanical parameters showed marked deviations from the distributions observed in the remaining asymptomatic cohort during the predefined quality-control procedure. Consequently, the final study cohort consisted of 90 healthy young adults, including 40 females (44.4%) and 50 males (55.6%), whose bilateral plantar pressure recordings were included in the statistical analyses. The study workflow is illustrated in Figure 1.

### 2.2. Study Population

The study population was selected to obtain a homogeneous cohort suitable for establishing a platform-specific reference dataset for dynamic plantar pressure assessment [17].

Participants were eligible for inclusion if they were aged 18–40 years, were able to walk independently without assistive devices, and had no known musculoskeletal, orthopedic, neurological, or other medical conditions that could influence gait or plantar pressure distribution. All participants were recruited from the Faculty of Physical Education and Sports and underwent standardized dynamic pedobarographic assessment. Participants represented a convenience sample of healthy, physically active university students. They were also required to have an absence of clinically evident lower-limb length discrepancy on physical examination, confirmed by clinical examination, and a physiologically normal gait pattern verified during the initial clinical assessment by a physician specialized in Physical and Rehabilitation Medicine. Participants were excluded if they had a history of recent lower-limb trauma, previous lower-extremity surgery, congenital or acquired foot deformities, pain during walking, or any medical condition known to affect gait performance or interfere with plantar pressure measurements. Individuals unable or unwilling to comply with the assessment protocol were also excluded.

The selected age range corresponds to a period of mature and stable gait patterns, minimizing the influence of developmental and age-related biomechanical changes and providing an appropriate population for the establishment of a reference dataset for dynamic plantar pressure assessment [18,19], thereby reducing potential sources of physiological gait variability.

Before enrolment, all participants underwent standardized clinical screening that included medical history, physical examination, lower-limb evaluation, and observational gait assessment to confirm eligibility and ensure the absence of conditions that could influence plantar loading. Body height and body weight were measured using standardized procedures, and body mass index (BMI) was calculated as weight (kg)/height^2^ (m^2^). This screening procedure contributed to the homogeneity of the study population and reduced inter-individual variability unrelated to normal gait characteristics.

### 2.3. Anthropometric Assessment

Height and body height were measured without footwear using a calibrated stadiometer and recorded to the nearest 0.1 cm. Body weight was measured using a calibrated digital scale, with participants wearing light clothing and no footwear, and recorded to the nearest 0.1 kg. Body mass index (BMI) was calculated as body weight in kilograms divided by height in meters squared (kg/m^2^).

### 2.4. Ethics Approval

The study was conducted in accordance with the ethical principles of the Declaration of Helsinki and relevant Good Clinical Practice guidelines. Ethical approval was obtained from the institutional Ethics Committee (approval no. 19/9 February 2021).

### 2.5. Dynamic Pedobarographic Assessment

Dynamic plantar pressure assessment was performed using a plantar pressure distribution platform (Footscan Scientific Version, RSscan International, Olen, Belgium) operating at a sampling frequency of 200 Hz and dedicated exclusively to dynamic measurements [20]. Before each testing session, the proper functioning of the RSscan platform and the validity of the calibration settings were verified according to the manufacturer’s recommendations. Immediately after each acquisition, the automatically generated plantar region segmentation was visually inspected by the same examiner. Trials showing incorrect regional segmentation, incomplete foot contact, or obvious acquisition artifacts were discarded and repeated until a valid recording was obtained.

To ensure natural walking conditions, the pressure platform was embedded within a 6 m walkway and covered with a thin ethylene-vinyl acetate (EVA) layer to minimize visual targeting and provide a comfortable walking surface. All participants walked barefoot at a self-selected comfortable speed.

Plantar pressure data were collected using the single-step protocol, which has been shown to provide reliable dynamic plantar pressure measurements while minimizing foot-targeting effects during gait. Each participant performed between three and five walking trials. The assessment was terminated as soon as three valid trials had been obtained; if fewer than three valid trials were achieved initially, additional trials were performed, up to a maximum of five attempts. A trial was considered valid when it demonstrated complete foot contact within the measurement area, stable heel strike, correct foot placement, total foot contact time between 600 and 800 ms, absence of technical acquisition errors, and reproducible plantar pressure measurements across repeated trials. Three valid recordings were obtained for each participant, providing three valid measurements for both the left and right foot. Participant-level values used for statistical analysis were calculated as the average of these three valid recordings. A trial was considered valid when it demonstrated stable heel strike and reproducible plantar pressure measurements. The average values obtained from all valid trials were used for subsequent analyses.

To allow bilateral assessment, plantar pressure recordings were obtained from both feet during consecutive gait cycles with alternating initial foot contact. Participants started walking approximately 3 m before reaching the pressure platform to ensure natural foot placement. Walking speed was not measured directly. To minimize excessive variability in gait performance while preserving a natural self-selected walking speed, total foot contact time was monitored during data acquisition and a predefined contact time interval of 600–800 ms was used as a quality-control criterion for accepting valid trials.

**Figure 2:**
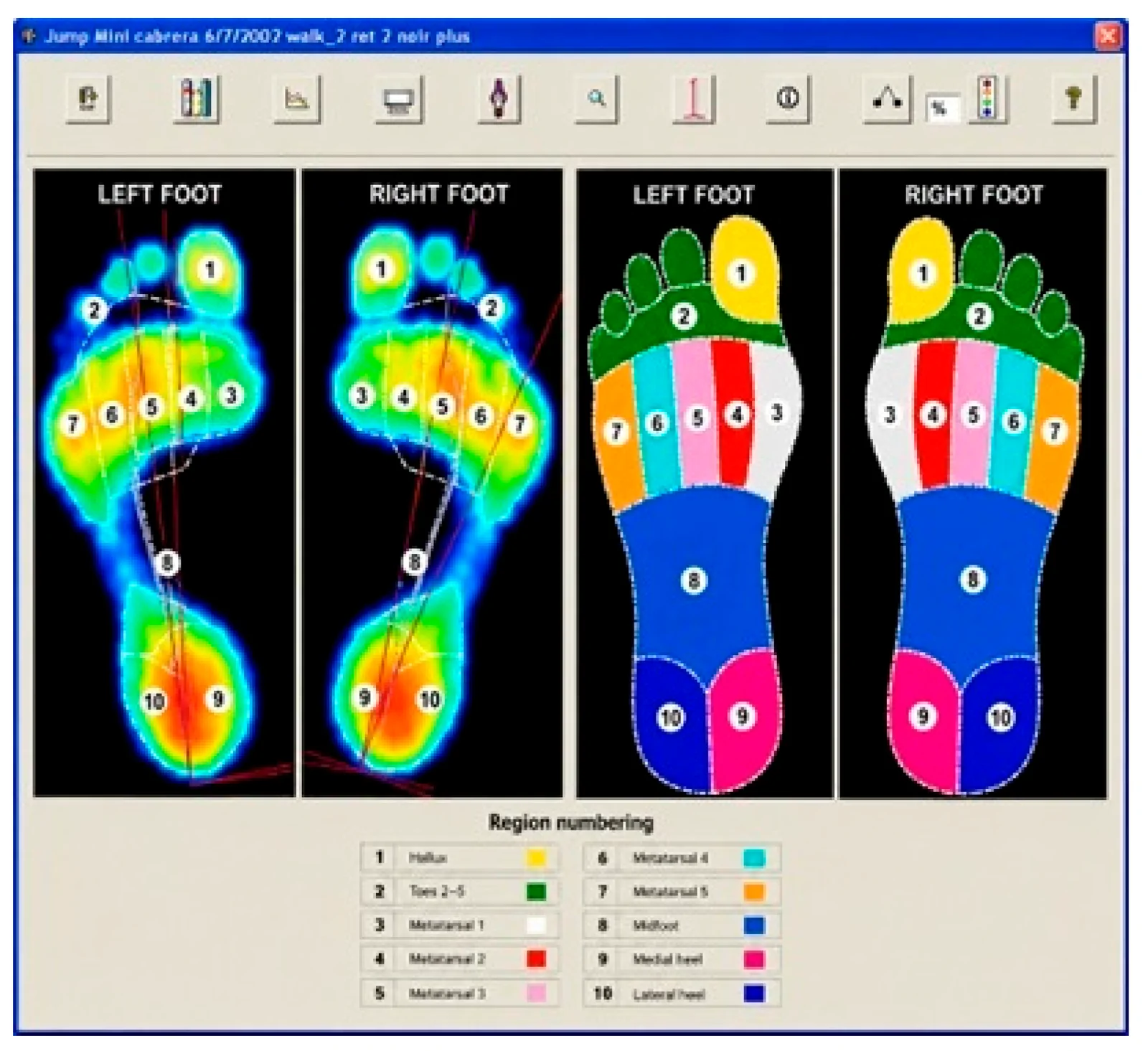
Anatomical segmentation of the plantar surface into ten predefined regions used for dynamic plantar pressure analysis. The plantar surface was divided into the following anatomical regions: (1) Hallux; (2) Toes 2–5; (3) Metatarsal 1; (4) Metatarsal 2; (5) Metatarsal 3; (6) Metatarsal 4; (7) Metatarsal 5; (8) Midfoot; (9) Medial heel; and (10) Lateral heel. The same regional segmentation was applied to both feet for all plantar pressure measurements.

The primary descriptive plantar loading parameters included maximum force (MaxF), impulse, and contact area. Load rate was additionally analyzed as a biomechanical parameter in the variability analysis. Maximum force (MaxF, N) was defined as the highest force recorded within each plantar region during the stance phase. Impulse (Ns) represented the time integral of force over the contact period. Load rate (N/s) represented the rate of force application. Contact area (cm^2^) corresponded to the plantar surface area in contact with the pressure platform. Measurements were obtained for ten predefined plantar regions (Figure 2). This standardized segmentation enabled detailed regional analysis of plantar loading during the stance phase of gait and served as the basis for establishing the proposed clinical reference values.

All plantar pressure recordings were acquired under identical testing conditions using the same equipment, standardized protocol, and evaluation procedure. Quality-control procedures included verification of complete foot contact, correct regional segmentation, absence of technical acquisition errors, stable heel strike, correct foot placement, total foot contact time between 600 and 800 ms, and reproducibility across repeated trials. Only recordings fulfilling all predefined quality-control criteria were included in the statistical analysis. Bilateral symmetry was assessed by comparing corresponding plantar pressure parameters between the left and right feet using paired Student’s *t*-tests.

### 2.6. Statistical Analysis

All statistical analyses were performed using Microsoft Excel (Microsoft 365, Microsoft Corp., Redmond, WA, USA) and IBM SPSS Statistics version 26.0 (IBM Corp., Armonk, NY, USA).

Descriptive statistics were calculated for the primary reference parameters (maximum force, impulse, and contact area), whereas load rate was additionally evaluated in the variability analysis. Continuous variables are presented as the mean, standard deviation (SD), minimum, maximum, and observed range.

To evaluate the inter-individual variability of the plantar loading parameters, the coefficient of variation (CV) was calculated for each plantar region and parameter. According to the observed CV values, distributions were classified as homogeneous (CV < 10%), relatively homogeneous (CV = 10–20%), or heterogeneous (CV > 20%).

The sex- and side-specific reference dataset was established using the observed mean, standard deviation, and range for each parameter, calculated separately for male and female participants and for the left and right feet.

Left–right comparisons were performed by comparing corresponding plantar loading parameters between the left and right feet using paired Student’s *t*-tests. These analyses were intended to identify systematic mean side differences at the group level and were not interpreted as tests of bilateral equivalence or individual symmetry. No predefined threshold for classifying symmetry or asymmetry was applied.

Sex-related differences in plantar loading parameters were evaluated separately for the left and right feet, with the participant considered as the statistical unit. Participant-level values represented the average of the three valid recordings obtained for each foot. Comparisons between female and male participants were performed using Welch’s independent-samples *t*-test, thereby avoiding the assumption of equal variances between groups. Given the moderate subgroup sizes, Welch’s *t*-test was considered a robust approach for the between-group comparisons because it does not require equal variances and is generally less sensitive to moderate departures from normality. Formal distributional normality testing was not available for all participant-level variables; this limitation is now explicitly acknowledged. Results were expressed as mean differences (male minus female), with corresponding 95% confidence intervals, two-sided *p*-values, and Hedges’ g effect sizes with 95% confidence intervals. Two-sided *p*-values were calculated for all comparisons; for readability, values below 0.001 are reported as *p* < 0.001 in the table. To account for multiple comparisons across plantar regions, parameters, and sides, the Benjamini–Hochberg false discovery rate (FDR) procedure was applied to the 60 sex-related comparisons. Statistical significance for these comparisons was defined as an FDR-adjusted *p*-value < 0.05.

Sex-related comparisons were not adjusted for anthropometric characteristics. Accordingly, differences in absolute maximum force between female and male participants were interpreted as unadjusted between-group differences and not as independent effects of sex, given the observed differences in body mass and height between the groups.

As this was a descriptive cross-sectional study designed to establish a reference dataset for dynamic plantar loading assessment rather than to test a predefined hypothesis, no formal a priori sample-size calculation was performed. The final sample consisted of all eligible participants who fulfilled the predefined inclusion criteria, completed the assessment protocol, and met the data-screening requirements.

## 3. Results

### 3.1. Demographic and Anthropometric Characteristics

Following the data-screening procedure described in Section 2.1, 12 participants (7 females and 5 males) were excluded because one or more recorded biomechanical parameters showed marked deviations from the distributions observed in the remaining asymptomatic cohort. Consequently, the final analysis included 90 healthy young adults (40 females and 50 males). Bilateral dynamic plantar pressure recordings were obtained from all included participants using the standardized pedobarographic assessment protocol. The demographic and anthropometric characteristics of the study population are presented in Table 1.

The final study cohort included 90 healthy young adults, with an overall mean age of 22.73 years. Female participants were slightly older than male participants (23.50 ± 2.08 vs. 22.11 ± 2.10 years). Male participants were taller and heavier than female participants, whereas mean BMI values were within the normal range in both groups. Overall, the cohort represented a young and physically active population.

### 3.2. Sex-Specific Reference Values for Dynamic Plantar Pressure Parameters

Sex- and side-specific reference values for the principal dynamic plantar pressure parameters are presented in Table 2. Mean values, standard deviations, and observed ranges were calculated separately for male and female participants and for the left and right feet.

Across both sexes, maximum force was greatest in the medial and lateral heel regions, followed by the central metatarsal regions (Metatarsal 2–Metatarsal 4), reflecting the physiological progression of load transfer during the stance phase of gait. Impulse exhibited a similar regional distribution, with the highest cumulative loading occurring at the heel and central forefoot. Contact area was greatest in the midfoot, whereas smaller contact areas were observed across the forefoot, heel, and toe regions.

The regional plantar loading pattern was highly consistent between the left and right feet and remained comparable between male and female participants. Although male participants tended to exhibit higher absolute values for several force-, impulse-, and contact area-related parameters, the overall regional pattern of plantar loading was broadly similar between sexes. These findings provide a sex- and side-specific, platform-specific reference dataset for dynamic plantar loading assessment in healthy young adults.

### 3.3. Inter-Individual Variability of Dynamic Plantar Loading Parameters

Inter-individual variability in maximum force, impulse, and contact area was assessed using the coefficient of variation (CV), calculated separately according to sex and side. The numerical CV values for each plantar region are presented in Table 3.

For descriptive interpretation of inter-individual variability, CV < 10% was considered homogeneous, CV = 10–20% relatively homogeneous, and CV > 20% heterogeneous. CV values represent inter-individual dispersion and should not be interpreted as measures of measurement reliability or reproducibility. Maximum force showed the greatest inter-individual variability among the three parameters. CV values exceeded 20% in most plantar regions and sex–side combinations, particularly in the toes, metatarsal regions, and midfoot. The highest variability was observed in the female group at the toes 2–5 and metatarsal regions, with CV values reaching 65.2% for the right toes 2–5 and 66.2% for the right first metatarsal region. Lower variability was observed in the heel regions, particularly among female participants, where CV values ranged from 15.5% to 19.3% for the medial and lateral heel, except for the male heel regions, where values were approximately 20–22%. Impulse demonstrated lower and more regionally consistent inter-individual variability than maximum force. Most CV values were within or close to the 10–20% range, although values slightly above 20% were observed in selected regions. Particularly low variability was observed for impulse at the first and second metatarsal regions in male participants, with CV values ranging from 5.1% to 7.2%. Contact area showed the lowest overall inter-individual variability. CV values were below 20% across all plantar regions and sex–side combinations, ranging from 7.7% to 19.4%. Values below 10% were observed in several metatarsal and heel regions, particularly among male participants. Overall, these findings indicate that inter-individual variability was parameter- and region-dependent, with maximum force exhibiting the greatest relative dispersion and contact area displaying the lowest relative dispersion within the study population.

### 3.4. Force Sex-Related Differences in Dynamic Plantar Loading Parameters

Sex-related differences in plantar loading parameters were evaluated separately for the left and right feet. Complete inferential results, including mean differences with 95% confidence intervals, Welch’s *t*-test statistics, FDR-adjusted *p*-values, and Hedges’ g effect sizes, are presented in Table 4.

For maximum force, significant sex-related differences after FDR correction were observed predominantly in the metatarsal and heel regions. Male participants showed higher values at Metatarsal 1 bilaterally, Metatarsal 3 bilaterally, Metatarsal 4 bilaterally, Metatarsal 5 bilaterally, the midfoot bilaterally, and the medial heel bilaterally (all FDR-adjusted *p* < 0.05). No significant sex-related differences were identified for the hallux, toes 2–5, Metatarsal 2, or lateral heel on either side.

For impulse, significant differences were observed at toes 2–5 on the right foot, Metatarsal 1 bilaterally, Metatarsals 2–4 bilaterally, the midfoot bilaterally, and the medial heel bilaterally (all FDR-adjusted *p* < 0.05), with higher values in male participants. No significant differences were observed for hallux, Metatarsal 5, or lateral heel on either side, or for toes 2–5 on the left foot.

Contact area showed the most consistent sex-related differences. Male participants demonstrated significantly greater contact area across all evaluated plantar regions and on both sides after FDR correction (all FDR-adjusted *p* < 0.05). Effect sizes for several of these differences were large, particularly in the metatarsal and heel regions.

Overall, sex-related differences were region- and parameter-dependent, with the most consistent differences observed for contact area, whereas maximum force and impulse showed more selective regional patterns.

### 3.5. Left–Right Comparison of Dynamic Plantar Loading Parameters

**Figure 3:**
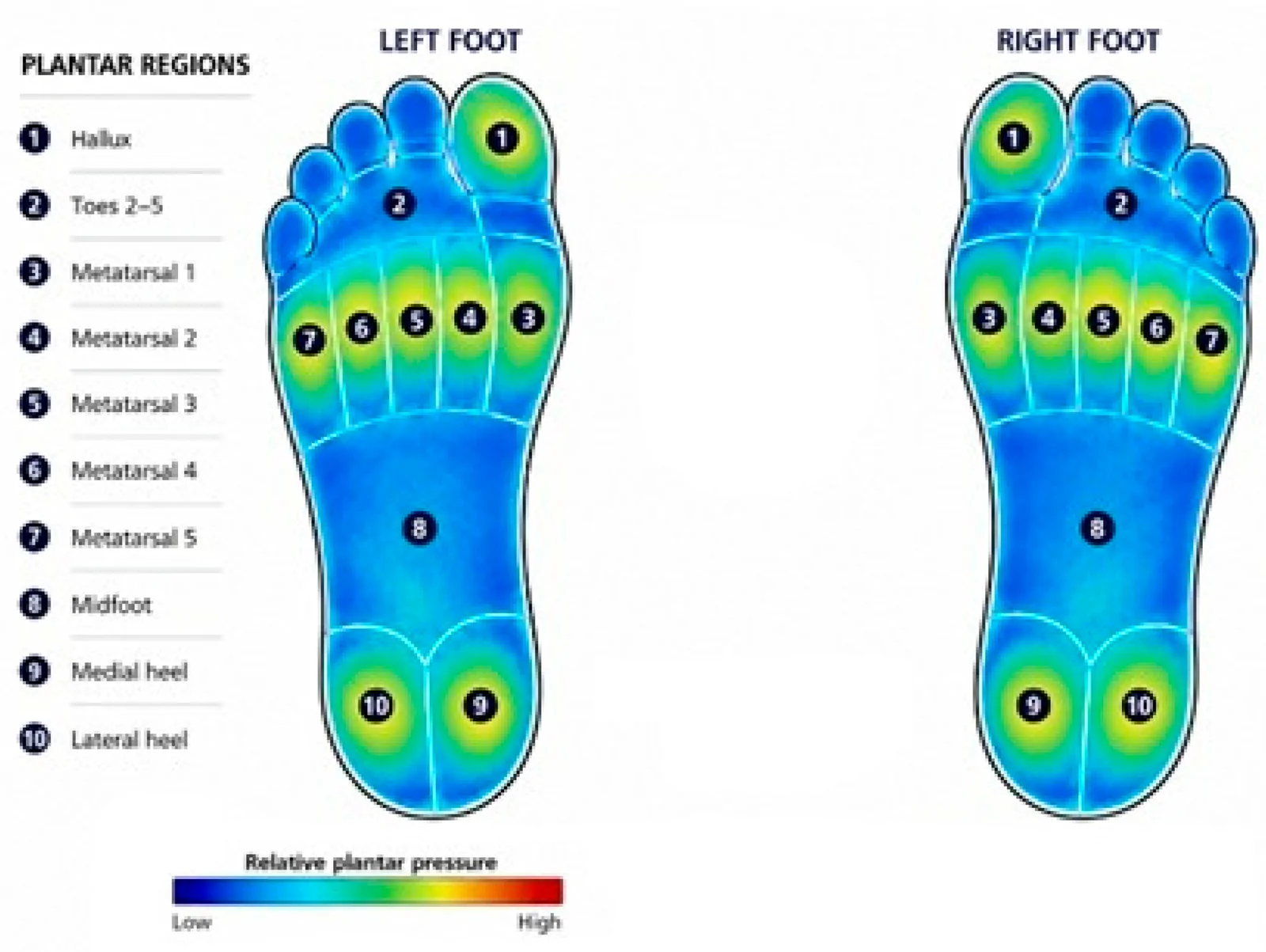
Representative bilateral plantar loading pattern in a healthy young adult. The plantar pressure map illustrates the anatomical distribution of loading across the predefined plantar regions of the left and right feet during dynamic gait assessment. Colors represent relative plantar pressure, ranging from low (blue) to high (red). The image is provided for illustrative purposes and does not represent a quantitative assessment of bilateral symmetry or equivalence within the study cohort.

Corresponding dynamic plantar loading parameters of the left and right feet were compared using paired Student’s *t*-tests. No statistically significant mean side differences were detected for the evaluated plantar loading parameters (all *p* > 0.05). These findings indicate the absence of statistically significant systematic directional differences at the group level and should not be interpreted as evidence of individual bilateral symmetry or equivalence (Figure 3).

## 4. Discussion

The present study provides platform- and population-specific descriptive plantar loading data obtained using the RSscan platform in a healthy young adult population. Beyond establishing reference values, these findings contribute to the objective biomechanical assessment of musculoskeletal function during gait [21,22]. Dynamic plantar pressure analysis reflects the integrated interaction between the musculoskeletal and neuromuscular systems, providing clinically relevant information on plantar loading patterns, gait biomechanics, and functional movement [21,22,23]. Consequently, normative plantar pressure datasets represent an essential foundation for interpreting pathological gait patterns and supporting clinical decision-making in rehabilitation, orthopedics, sports medicine, and neurological disorders [22,23,24].

The principal findings of the present study were the identification of consistent regional plantar loading patterns across the healthy adult cohort, parameter- and region-dependent differences between male and female participants, and the absence of statistically significant mean side differences between the left and right feet. The regional plantar loading pattern observed in this cohort was characterized by higher maximum force and impulse values in the heel and central metatarsal regions, whereas contact area was greater in the midfoot and heel regions. This spatial distribution is compatible with previously described patterns of plantar loading during physiological gait; however, the present regional summary measures do not permit direct characterization of the temporal progression of load transfer during stance. The relatively narrow age range and the recruitment of healthy, physically active young adults reduced the influence of broad age-related variability. However, the observed sex-related differences in age and anthropometric characteristics should be considered when interpreting the sex-specific plantar loading patterns. These characteristics support the suitability of the investigated population for generating clinical reference values specific to healthy, physically active young adults.

The interpretation of plantar pressure data must nevertheless remain closely linked to the equipment and protocol used. Ramanathan et al. [25] demonstrated acceptable repeatability of plantar pressure measurements obtained using the Pedar-X system and emphasized the importance of standardized acquisition procedures. In addition, differences between plantar pressure measurement systems may limit direct comparison of values obtained with different devices [14]. Consequently, the values reported in the present study should be considered platform- and protocol-specific clinical reference values rather than universally transferable normative thresholds.

Regarding sex-related differences, the present analysis demonstrated parameter- and region-dependent differences between male and female participants. After correction for multiple comparisons, differences in maximum force and impulse were predominantly observed in selected metatarsal, midfoot, and heel regions, whereas contact area showed the most consistent between-group differences across plantar regions. These findings support the use of sex-specific descriptive data when interpreting plantar loading patterns. Previous studies have similarly reported sex-related differences in pedobarographic parameters among healthy adults [26,27,28], while broader evidence indicates that plantar loading outcomes are influenced by population characteristics and anthropometric profile [9]. These between-group differences should, however, be interpreted cautiously. Male participants were taller and heavier than female participants, and the present comparisons were not adjusted for body mass or other anthropometric characteristics. Consequently, differences in absolute maximum force cannot be attributed independently to sex and may partly reflect differences in body size. The observed findings should therefore be interpreted as unadjusted differences between the male and female groups rather than independent sex effects. Future studies should evaluate body-mass-normalized loading variables and/or multivariable models incorporating relevant anthropometric characteristics. Walking speed may also contribute to between-participant and between-group differences in plantar loading [28,29]. In the present study, participants walked at a self-selected pace, while total contact time was used only as an acquisition quality-control criterion. Because walking velocity was not directly measured, its potential contribution to the observed sex-related differences cannot be excluded.

A second major finding was that inter-individual variability differed according to both the plantar region and the biomechanical parameter evaluated. Maximum force showed the greatest relative dispersion, with CV values exceeding 20% in most plantar regions and sex–side combinations, particularly in the toes, metatarsal regions, and midfoot. Impulse demonstrated lower and more regionally consistent variability, with most CV values within or close to the 10–20% range, although values above 20% were observed in selected regions. Contact area showed the lowest overall relative dispersion, with CV values remaining below 20% across all plantar regions and sex–side combinations. These findings indicate that inter-individual variability is parameter- and region-dependent and should therefore be considered when interpreting the proposed reference dataset. Previous methodological studies have also demonstrated that the performance of plantar loading measurements may vary according to the parameter and anatomical region examined. Xu et al. [20] reported that the Footscan system can provide reliable dynamic plantar pressure data, although repeatability varies across anatomical regions and measured variables. Gurney et al. [30] similarly observed good-to-excellent test–retest reliability for several barefoot plantar pressure parameters in healthy adults. Although measurement reliability and inter-individual variability represent distinct concepts, these findings reinforce the importance of considering both the parameter evaluated and its anatomical location when interpreting pedobarographic data. In the present study, contact area showed comparatively lower inter-individual variability, whereas maximum force exhibited greater relative dispersion across several plantar regions. Maximum force showed particularly marked regional variability. The greatest relative variability was observed in the toes, metatarsal regions, and midfoot, whereas comparatively lower CV values were generally observed in the heel regions, particularly among female participants. The hallux nevertheless showed CV values slightly above 20% across all sex–side combinations. Similar region-dependent variability of plantar pressure has recently been reported, indicating that different plantar regions exhibit distinct biomechanical variability profiles that should be considered when interpreting pedobarographic data [31]. The higher variability of maximum force may plausibly be influenced by inter-individual differences in foot morphology, limb dominance, foot placement, and gait strategy, as suggested by previous biomechanical studies [25,30,32,33]. However, these potential determinants were not directly quantified in the present study, and their contribution to the observed variability cannot be established from the current data. Even under standardized conditions, plantar force distribution remains sensitive to individual biomechanical characteristics and methodological factors influencing gait assessment [25,30,32,33]. Accordingly, isolated maximum-force values should not be interpreted without considering the corresponding anatomical region, sex, body dimensions, acquisition protocol, and the distribution of the remaining plantar pressure parameters [25,32,33].

The left–right comparison represented an additional component of the present analysis. No statistically significant mean side differences were detected between corresponding plantar loading parameters of the left and right feet. This finding indicates the absence of statistically significant systematic directional differences at the group level but should not be interpreted as evidence of bilateral equivalence or individual symmetry. A paired *t*-test evaluates whether the mean directional difference between paired measurements differs from zero and may therefore conceal participant-specific side-to-side differences. The eligibility criterion concerning lower-limb length was intended to exclude clinically evident limb-length discrepancy as a potential confounder of gait and plantar loading and did not constitute a criterion of plantar loading symmetry. Nevertheless, the selection of a healthy cohort without clinically evident structural asymmetry should be considered when interpreting the left–right comparisons. Previous studies in healthy populations have likewise reported relatively comparable plantar loading patterns between the two feet, although measurable regional side-to-side differences may occur [20,30,32]. These observations are broadly consistent with the absence of systematic mean side differences observed in the present cohort, while also emphasizing that group-level similarity does not necessarily imply individual bilateral symmetry. Participant-specific side-to-side differences may arise from limb dominance, habitual movement strategies, anatomical variation, or natural step-to-step fluctuations. Previous biomechanical studies have similarly shown that measurable side-to-side differences may occur in healthy individuals and that gait symmetry is influenced by several functional and methodological factors. Riskowski et al. [32] emphasized that the functional significance of bilateral asymmetry depends on its magnitude and on the characteristics of the population examined, while Patterson et al. [33] demonstrated that gait symmetry and walking velocity represent related but distinct constructs. Similarly, Kowalski et al. [34] reported that different limb-matching approaches generally produced comparable statistical outcomes in healthy gait, with a significant limb-matching effect identified for only one of the 25 biomechanical variables examined. These findings support the view that systematic side-related effects may be limited in healthy gait, although they do not establish individual bilateral equivalence. Future studies should therefore complement group-level paired comparisons with participant-level absolute side-to-side differences, explicitly defined symmetry metrics, confidence intervals, and agreement or equivalence approaches. Such analyses would allow a more rigorous distinction between the absence of a systematic directional difference and true bilateral agreement. Nevertheless, side-to-side differences in plantar loading should be interpreted within the broader clinical context, considering anthropometric characteristics, gait pattern, and the specific plantar loading parameters evaluated, rather than as an isolated indicator of normality or pathology.

The present study contributes sex- and side-specific clinical reference values obtained under standardized conditions using the RSscan platform. The availability of comparable reference data remains limited, and values reported in one population or obtained using one measurement system cannot necessarily be transferred directly to another. Koo et al. [26] emphasized the importance of population-specific pedobarographic data, while Telfer and Bigham [9] demonstrated that both population characteristics and measurement systems substantially influence barefoot plantar pressure outcomes. The present dataset may therefore serve as a benchmark for studies using a comparable population, device, segmentation model, and acquisition protocol.

To further contextualize the present dataset, Table 5 summarizes selected findings from previous studies of plantar loading in healthy adults alongside the findings of the present study. Direct numerical comparison was restricted to parameters and anatomical regions with sufficient methodological comparability. Differences in measurement systems, acquisition protocols, segmentation models, parameter definitions, and population characteristics should therefore be considered when interpreting differences across studies.

The comparison highlights both similarities and methodological differences across published datasets. Xu et al. [20], using the Footscan platform and an equivalent ten-region segmentation model, similarly identified the medial heel as the region with the highest maximum force and the midfoot as the region with the greatest contact area. In contrast, studies examining sex-related differences using different outcomes and measurement systems have produced variable findings. Koo et al. [26] reported higher forefoot peak pressures in men, whereas Yamamoto et al. [27] reported higher peak pressures in several regions in women. These apparently divergent findings should not be considered directly contradictory, because peak pressure and maximum force represent different biomechanical quantities and are influenced by measurement system, segmentation, protocol, walking conditions, and anthropometric characteristics. Furthermore, Leyh and Feipel [28] demonstrated a significant influence of walking velocity on several plantar pressure parameters. Collectively, these comparisons reinforce the need to interpret the present values as platform-, protocol-, and population-specific descriptive benchmarks rather than universally interchangeable normative values.

From a clinical perspective, parameters exhibiting lower inter-individual variability may provide useful complementary information when interpreting plantar loading during functional assessment. Contact area showed comparatively low relative dispersion across the investigated plantar regions, whereas maximum force should be interpreted more cautiously because of its greater regional and inter-individual variability. Consistent with previous methodological studies, interpretation of pedobarographic data is strengthened by integrating multiple complementary parameters rather than relying on a single measurement [20,25,30,35]. The reference values established in the present study may contribute to this objective approach by providing normative data for healthy young adults. They may also serve as a baseline for future comparisons involving individuals with musculoskeletal, neurological, metabolic, or post-traumatic impairments. However, the present cross-sectional study did not evaluate diagnostic accuracy, responsiveness to intervention, or the ability of the proposed values to differentiate healthy from pathological populations. Therefore, their clinical utility for early detection, rehabilitation monitoring, and treatment-response assessment requires prospective validation in relevant patient groups.

Several limitations should be acknowledged. First, participants were recruited from a single Faculty of Physical Education and Sports and represented a convenience sample of young, physically active adults. This limits the generalizability of the findings to sedentary individuals, older adults, children, and populations with different anthropometric or activity profiles. Although participants were described as physically active, their activity level and type of sports participation were not quantified using a standardized instrument. The participants covered an age range of 18–40 years, but age-related trends within this interval were not specifically analyzed and should be investigated in future studies. Differences in height and body mass between male and female participants may have contributed to the observed between-group differences in plantar loading. Because the analyses were not adjusted for anthropometric characteristics and absolute maximum force was not normalized to body mass, these findings cannot distinguish an independent sex effect from the influence of body size.

Second, all measurements were obtained using a single plantar pressure platform and a specific regional segmentation model. The plantar region segmentation used in this study was predefined by the RSscan analysis software and reflects a standardized regional masking approach widely used for dynamic pedobarographic assessment. Although combining toes 2–5 and treating the midfoot as a single anatomical region may reduce regional anatomical resolution, this approach ensures methodological consistency and facilitates comparison among participants evaluated using the same platform. Future studies employing higher-resolution masking protocols may provide additional clinically relevant information regarding regional loading patterns. An additional limitation concerns the assessment of left–right differences. The paired *t*-test used in the present study evaluates systematic mean directional differences at the group level but does not establish bilateral equivalence or quantify participant-specific asymmetry. Individual absolute side-to-side differences, dedicated symmetry indices, and agreement or equivalence analyses were not available. Consequently, the present findings should not be interpreted as demonstrating bilateral symmetry. Third, walking speed was not measured directly in the present study and was controlled only indirectly through a predefined total foot contact time interval during data acquisition. Although this approach contributed to reducing excessive gait variability while preserving self-selected walking, total foot contact time cannot be considered equivalent to walking speed and may itself be influenced by anthropometric characteristics, gait strategy, and sex. Consequently, residual variability related to walking speed may have influenced plantar loading patterns and should be considered when interpreting the proposed reference dataset and sex-specific comparisons. Future studies should incorporate direct measurements of walking speed to further improve methodological standardization.

Fourth, although repeated trials and predefined quality-control criteria were used, dynamic plantar pressure measurements remain sensitive to foot placement, step-to-step variation, and experimental conditions. The RSscan platform also does not provide information regarding joint kinematics, muscle activation, or proximal lower-limb mechanics, and time-series loading trajectories were not analyzed in the present study. Consequently, the physiological mechanisms underlying the observed regional plantar loading patterns, including temporal load transfer and foot rollover, cannot be directly inferred from the present measurements. Finally, the proposed clinical reference values were based on observed means, standard deviations, and ranges. They should not be interpreted as formal population reference intervals based on representative sampling and percentile-derived limits.

The standardized eligibility screening, repeated valid trials, uniform acquisition conditions, and use of the same equipment and regional segmentation model for all participants strengthened the internal validity of the study. However, residual variability related to self-selected walking speed, foot placement, and step-to-step fluctuations cannot be entirely excluded. External validity is limited by the single-center convenience sampling of young, physically active adults and by the use of one specific pedobarographic system and protocol. Consequently, the proposed reference values should be interpreted as population- and platform-specific and require validation in broader age groups, sedentary populations, clinical cohorts, and other measurement systems. The growing integration of quantitative gait analysis into rehabilitation medicine highlights the need for robust, standardized reference values that facilitate the distinction between physiological variability and clinically meaningful biomechanical alterations [36].

Overall, the findings indicate that healthy young adults demonstrate characteristic regional plantar loading patterns and parameter- and region-dependent differences between male and female participants. No statistically significant mean side differences were detected between the left and right feet; however, this finding should not be interpreted as evidence of individual bilateral symmetry or equivalence. Inter-individual variability was parameter- and region-dependent, with contact area showing comparatively lower relative dispersion and maximum force showing greater variability, particularly in the forefoot and midfoot regions. This study provides a platform- and population-specific reference dataset that may support the interpretation of dynamic plantar loading assessments, while highlighting the importance of considering sex, anthropometric characteristics, anatomical region, parameter type, and measurement protocol when interpreting physiological variability.

## 5. Conclusions

This study established a platform-specific descriptive reference dataset comprising sex- and side-specific values for maximum force, impulse, and contact area in healthy young adults assessed using the RSscan platform under standardized conditions. The findings demonstrated characteristic regional plantar loading patterns, parameter- and region-dependent differences between male and female participants, and no statistically significant mean side differences between the left and right feet.

The proposed descriptive values may provide a useful platform- and protocol-specific benchmark for the interpretation of pedobarographic measurements obtained under comparable assessment conditions and for future research involving healthy and clinical populations. Contact area exhibited the lowest inter-individual variability across anatomical regions, whereas maximum force demonstrated greater regional variability and should therefore be interpreted in conjunction with complementary plantar loading parameters. Further validation in larger and more diverse populations and across different plantar pressure measurement systems and protocols is required before these values can be considered broadly applicable clinical reference values.

## Figures and Tables

**Table 1:** Anthropometric characteristics.

Characteristics	Female (n = 40) Mean (SD)	Male (n = 50) Mean (SD)	Overall Mean
Age	23.50 (2.08)	22.11 (2.10)	22.73 (2.19)
Height (m)	1.64 (0.06)	1.81 (0.04)	1.73 (0.10)
Weight (kg)	53.24 (4.10)	71.80 (6.93)	63.55 (10.59)
BMI (kg/m^2^)	19.14 (1.76)	20.70 (1.79)	20.01 (1.93)

Variables are reported as mean and SD = standard deviation; BMI = body mass index.

**Table 2:** Sex- and side-specific reference values for the principal dynamic plantar pressure parameters in healthy young adults.

Parameter/ Plantar Region	Male-Left	Male-Right	Female-Left	Female-Right
Maximum force (N)				
Hallux	194.5 ± 45.4 [123.5–239.7]	194.2 ± 45.9 [113.1–235.3]	199.6 ± 44.1 [128.8–235.9]	183.7 ± 41.9 [123.4–231.8]
Toes 2–5	42.7 ± 19.3 [21.5–97.3]	43.8 ± 23.9 [31.9–96.3]	36.3 ± 19.3 [18.6–68.6]	37.6 ± 24.5 [12.3–73.2]
Metatarsal 1	120.6 ± 48.6 [99.3–234.0]	136.6 ± 51.1 [107.6–225.0]	72.4 ± 39.6 [28.5–145.8]	89.1 ± 59.0 [39.6–157.7]
Metatarsal 2	201.2 ± 63.1 [107.9–321.7]	217.7 ± 70.8 [107.9–315.3]	201.8 ± 41.1 [169.2–258.7]	207.6 ± 47.4 [151.8–267.1]
Metatarsal 3	238.5 ± 70.2 [149.1–317.7]	228.1 ± 67.1 [103.3–312.0]	174.1 ± 59.7 [99.2–231.7]	187.4 ± 74.9 [76.5–248.8]
Metatarsal 4	203.4 ± 75.2 [139.4–314.4]	191.6 ± 60.0 [131.1–250.7]	121.5 ± 46.0 [72.3–212.2]	105.0 ± 49.6 [55.3–201.6]
Metatarsal 5	159.2 ± 60.0 [59.9–232.1]	146.7 ± 67.3 [77.5–231.0]	65.3 ± 29.8 [15.6–82.8]	40.2 ± 24.5 [18.2–87.5]
Midfoot	225.1 ± 99.8 [104.3–369.3]	214.4 ± 81.0 [102.0–322.7]	178.8 ± 51.8 [115.1–220.0]	173.7 ± 54.9 [124.5–233.6]
Medial Heel	340.6 ± 68.2 [268.2–426.6]	360.9 ± 72.5 [243.2–410.1]	288.4 ± 44.6 [223.7–338.4]	280.2 ± 56.7 [222.3–329.6]
Lateral Heel	276.7 ± 62.1 [185.0–337.3]	287.5 ± 64.0 [185.6–335.6]	270.9 ± 43.7 [223.4–326.1]	277.9 ± 44.0 [220.5–318.2]
Impulse (Ns)				
Hallux	42.0 ± 7.7 [35.4–51.0]	41.7 ± 8.4 [32.3–52.6]	40.6 ± 8.2 [33.0–61.1]	40.0 ± 8.2 [32.0–68.6]
Toes 2–5	7.9 ± 1.6 [6.2–10.8]	7.9 ± 1.6 [6.1–9.5]	7.5 ± 1.2 [4.2–14.3]	6.8 ± 1.2 [4.5–13.9]
Metatarsal 1	61.6 ± 4.2 [57.6–74.4]	61.0 ± 4.4 [56.9–74.6]	57.9 ± 8.6 [39.7–74.5]	47.1 ± 8.2 [36.6–72.4]
Metatarsal 2	70.5 ± 3.6 [67.9–82.2]	74.4 ± 4.4 [64.4–81.8]	48.0 ± 9.2 [28.4–64.9]	54.5 ± 9.8 [48.6–70.6]
Metatarsal 3	72.7 ± 13.8 [58.7–88.4]	71.5 ± 14.0 [58.2–86.1]	50.5 ± 10.3 [36.5–68.4]	54.0 ± 10.6 [40.9–68.6]
Metatarsal 4	63.4 ± 9.5 [54.5–73.6]	62.2 ± 9.7 [53.6–73.1]	56.0 ± 11.2 [32.2–67.3]	52.8 ± 9.0 [36.7–79.2]
Metatarsal 5	46.7 ± 9.2 [35.4–54.1]	45.7 ± 9.1 [31.4–53.8]	45.4 ± 10.1 [35.8–69.5]	46.3 ± 9.5 [41.9–66.5]
Midfoot	66.5 ± 12.4 [43.3–79.1]	66.7 ± 12.2 [41.8–79.7]	55.7 ± 10.7 [39.5–70.1]	56.2 ± 10.9 [38.2–71.8]
Medial Heel	86.4 ± 16.2 [69.5–102.4]	92.2 ± 14.6 [77.5–106.7]	73.4 ± 11.6 [56.7–81.3]	72.2 ± 12.2 [55.0–84.8]
Lateral Heel	74.0 ± 14.6 [59.8–90.2]	74.4 ± 14.4 [62.5–91.5]	69.0 ± 13.2 [58.3–89.9]	69.6 ± 13.6 [40.9–81.8]
Contact area (cm^2^)				
Hallux	20.5 ± 2.7 [18.1–23.8]	21.4 ± 3.4 [17.4–26.6]	17.4 ± 2.6 [14.8–20.0]	17.0 ± 2.4 [15.2–20.2]
Toes 2–5	15.1 ± 2.3 [13.4–18.3]	14.5 ± 2.4 [11.3–17.6]	12.3 ± 1.5 [10.5–15.5]	12.6 ± 1.4 [10.9–15.8]
Metatarsal 1	12.9 ± 2.0 [10.6–15.2]	18.5 ± 3.1 [15.4–21.8]	10.5 ± 1.8 [8.6–12.3]	12.8 ± 1.3 [9.8–13.4]
Metatarsal 2	12.4 ± 1.3 [9.0–15.6]	14.7 ± 1.3 [12.9–16.3]	10.3 ± 1.2 [8.1–12.0]	12.2 ± 1.3 [9.4–15.0]
Metatarsal 3	10.2 ± 1.0 [8.9–11.4]	11.6 ± 0.9 [10.8–13.9]	8.5 ± 1.0 [7.2–10.1]	9.8 ± 1.3 [7.5–12.2]
Metatarsal 4	10.6 ± 1.2 [7.9–13.2]	11.8 ± 1.1 [10.6–14.2]	9.0 ± 1.1 [6.8–10.6]	9.7 ± 1.0 [7.9–12.0]
Metatarsal 5	14.9 ± 1.9 [12.6–17.1]	11.9 ± 2.0 [9.9–14.5]	11.7 ± 1.8 [8.6–12.8]	10.1 ± 1.7 [8.1–13.5]
Midfoot	46.2 ± 7.8 [37.5–54.6]	40.8 ± 7.9 [31.2–49.5]	35.5 ± 4.7 [27.6–42.2]	33.8 ± 4.5 [30.5–41.4]
Medial Heel	20.7 ± 2.1 [17.9–24.8]	21.3 ± 1.9 [19.1–23.6]	16.8 ± 1.9 [13.1–19.9]	17.6 ± 2.1 [14.7–21.2]
Lateral Heel	17.8 ± 1.8 [15.5–20.1]	18.2 ± 1.4 [16.8–19.6]	14.2 ± 1.9 [10.5–17.6]	15.1 ± 1.8 [11.0–17.5]

Values are presented as mean ± standard deviation [observed minimum–maximum]. Impulse is expressed in Ns = Newton second, as specified in the original RSscan.

**Table 3:** Coefficients of variation (%) for sex- and side-specific plantar loading parameters.

Parameter/ Plantar Region	Male-Left CV (%)	Male-Right CV (%)	Female-Left CV (%)	Female-Right CV (%)
Maximum force				
Hallux	23.3	23.6	22.1	22.8
Toes 2–5	45.2	54.6	53.2	65.2
Metatarsal 1	40.3	37.4	54.7	66.2
Metatarsal 2	31.4	32.5	20.4	22.8
Metatarsal 3	29.4	29.4	34.3	40.0
Metatarsal 4	37.0	31.3	37.9	47.2
Metatarsal 5	37.7	45.9	45.6	60.9
Midfoot	44.3	37.8	29.0	31.6
Medial Heel	20.0	20.1	15.5	19.3
Lateral Heel	22.4	22.3	16.1	15.8
Impulse				
Hallux	18.3	20.1	20.2	20.5
Toes 2–5	20.3	20.3	16.0	17.4
Metatarsal 1	6.8	7.2	14.9	17.4
Metatarsal 2	5.1	5.9	19.2	18.0
Metatarsal 3	19.0	19.6	20.4	19.6
Metatarsal 4	15.0	15.6	20.0	17
Metatarsal 5	19.7	19.9	22.2	20.5
Midfoot	18.6	18.3	19.2	19.4
Medial Heel	18.7	15.8	15.8	16.9
Lateral Heel	19.7	19.4	19.1	19.5
Contact area				
Hallux	13.2	15.9	14.9	14.1
Toes 2–5	15.2	16.6	12.2	11.1
Metatarsal 1	15.5	16.8	17.1	10.2
Metatarsal 2	10.5	8.8	11.7	10.7
Metatarsal 3	9.8	7.8	11.8	13.3
Metatarsal 4	11.3	9.3	12.2	10.3
Metatarsal 5	12.8	16.8	15.4	16.8
Midfoot	16.9	19.4	13.2	13.3
Medial Heel	10.1	8.9	11.3	11.9
Lateral Heel	10.1	7.7	13.4	11.9

CV, coefficient of variation. Values are expressed as percentages and were calculated as CV = (SD/mean) × 100 from the sex- and side-specific descriptive statistics reported in Table 2.

**Table 4:** Sex-related differences in dynamic plantar loading parameters according to side.

Parameter/ Region	Side	Female Mean ± SD	Male Mean ± SD	Mean Difference (M-F) [95%CI]	Welch t (df)	*p*	FDR *p*	Hedges’ g [95%CI]
Maximum force (N)								
Hallux	Left	199.6 ± 44.1	194.5 ± 45.4	−5.1 [−23.9, 13.7]	−0.54 (84.7)	0.592	0.623	−0.11 [−0.53, 0.30]
Hallux	Right	183.7 ± 41.9	194.2 ± 45.9	10.5 [−7.9, 28.9]	1.13 (86.4)	0.261	0.307	0.24 [−0.18, 0.65]
Toes 2–5	Left	36.3 ± 19.3	42.7 ± 19.3	6.4 [−1.7, 14.5]	1.56 (83.7)	0.122	0.152	0.33 [−0.09, 0.74]
Toes 2–5	Right	37.6 ± 24.5	43.8 ± 23.9	6.2 [−4.0, 16.4]	1.21 (82.8)	0.231	0.278	0.25 [−0.16, 0.67]
Metatarsal 1	Left	72.4 ± 39.6	120.6 ± 48.6	48.2 [29.7, 66.7]	5.18 (88.0)	<0.001	<0.001	1.07 [0.62, 1.51]
Metatarsal 1	Right	89.1 ± 59.0	136.6 ± 51.1	47.5 [24.0, 71.0]	4.03 (77.6)	<0.001	<0.001	0.86 [0.43, 1.29]
Metatarsal 2	Left	201.8 ± 41.1	201.2 ± 63.1	−0.6 [−22.5, 21.3]	−0.05 (84.8)	0.957	0.957	−0.01 [−0.42, 0.40]
Metatarsal 2	Right	207.6 ± 47.4	217.7 ± 70.8	10.1 [−14.8, 35.0]	0.81 (85.5)	0.422	0.460	0.16 [−0.25, 0.58]
Metatarsal 3	Left	174.1 ± 59.7	238.5 ± 70.2	64.4 [37.2, 91.6]	4.70 (87.6)	<0.001	<0.001	097 [0.53, 1.41]
Metatarsal 3	Right	187.4 ± 74.9	228.1 ± 67.1	40.7 [10.5, 70.9]	2.68 (79.2)	0.009	0.012	0.57 [0.15, 0.99]
Metatarsal 4	Left	121.5 ± 46.0	203.4 ± 75.2	81.9 [56.3, 107.5]	6.36 (82.8)	<0.001	<0.001	1.27 [0.82, 1.72]
Metatarsal 4	Right	105.0 ± 49.6	191.6 ± 60.0	86.6 [63.6, 109.6]	7.49 (87.9)	<0.001	<0.001	1.54 [1.07, 2.01]
Metatarsal 5	Left	65.3 ± 29.8	159.2 ± 60.0	93.9 [74.6, 113.2]	9.67 (74.9)	<0.001	<0.001	1.90 [1.40, 2.40]
Metatarsal 5	Right	40.2 ± 24.5	146.7 ± 67.3	106.5 [86.0, 127.0]	10.36 (64.4)	<0.001	<0.001	2.40 [1.49, 2.51]
Midfoot	Left	178.8 ± 51.8	225.1 ± 99.8	46.3 [13.8, 78.8]	2.84 (76.6)	0.006	0.008	0.56 [0.14, 0.98]
Midfoot	Right	173.7 ± 54.9	214.4 ± 81.0	40.7 [12.1, 69.3]	2.83 (85.9)	0.006	0.008	0.57 [0.15, 0.99]
Medial Heel	Left	288.4 ± 44.6	340.6 ± 68.2	52.2 [28.4, 76.0]	4.37 (84.9)	<0.001	<0.001	0.88 [0.45, 1.31]
Medial Heel	Right	280.2 ± 54.1	360.9 ± 72.5	80.7 [54.2, 107.2]	6.04 (87.6)	<0.001	<0.001	1.23 [0.78, 1.68]
Lateral Heel	Left	270.9 ± 43.7	276,7 ± 62.1	5.8 [−16.4, 28.0]	0.52 (86.7)	0.605	0.626	0.11 [−0.31, 0.52]
Lateral Heel	Right	277.9 ± 44.0	287.5 ± 64.0	9.6 [−13.1, 32.3]	0.84 (86.2)	0.403	0.456	0.17 [−0.24, 0.58]
Impulse (Ns)								
Hallux	Left	40.6 ± 8.2	42.0 ± 7.7	1.4 [−2.0, 4.8]	0.83 (81.2)	0.411	0.456	0.18 [−0.24, 0.59]
Hallux	Right	40.0 ± 8.2	41.7 ± 8.4	1.7 [−1.8, 5.2]	0.97 (84.5)	0.336	0.388	0.20 [−0.21, 0.62]
Toes 2–5	Left	7.5 ± 1.2	7.9 ± 1.6	0.4 [−0.2, 1.0]	1.35 (87.7)	0.179	0.219	0.28 [−0.14, 0.69]
Toes 2–5	Right	6.8 ± 1.2	7.9 ± 1.6	1.1 [0.5, 1.7]	3.73 (87.7)	<0.001	<0.001	0.76 [0.33, 1.19]
Metatarsal 1	Left	57.9 ± 8.6	61.6 ± 4.2	3.7 [0.7, 6.7]	2.49 (53.7)	0.016	0.021	0.56 [0.14, 0.98]
Metatarsal 1	Right	47.1 ± 8.2	61.0 ± 4.4	13.9 [11.0, 16.8]	9.67 (56.6)	<0.001	<0.001	2.16 [1.64, 2.69]
Metatarsal 2	Left	48.0 ± 9.2	70.5 ± 3.6	22.5 [19.4, 25.6]	14.60 (48.6)	<0.001	<0.001	3.34 [2.69, 3.98]
Metatarsal 2	Right	54.5 ± 9.8	74.4 ± 4.4	19.9 [16.5, 23.3]	11.92 (51.5)	<0.001	<0.001	2.70 [2.13, 3.28]
Metatarsal 3	Left	50.5 ± 10.3	72.7 ± 13.8	22.2 [17.1, 27.3]	8.73 (87.6)	<0.001	<0.001	1.78 [1.29, 2.27]
Metatarsal 3	Right	54.0 ± 10.6	71.5 ± 14.0	17.5 [12.3, 22.7]	6.75 (87.8)	<0.001	<0.001	1.38 [0.92, 1.84]
Metatarsal 4	Left	56.0 ± 11.2	63.4 ± 9.5	7.4 [3.0, 11.8]	3.33 (76.6)	0.001	0.002	0.71 [0.29, 1.14]
Metatarsal 4	Right	52.8 ± 9.0	62.2 ± 9.7	9.4 [5.5, 13.3]	4.76 (86.0)	<0.001	<0.001	0.99 [0.55, 1.43]
Metatarsal 5	Left	45.4 ± 10.1	16.7 ± 9.2	1.3 [−2.8, 5.4]	0.63 (79.9)	0.530	0.568	0.13 [−0.28, 0.55]
Metatarsal 5	Right	46.3 ± 9.5	45.7 ± 9.1	−0.6 [−4.5, 3.3]	−0.30 (82.1)	0.762	0.775	−0.06 [−0.48, 0.35]
Midfoot	Left	55.7 ± 10.7	66.5 ± 12.4	10.8 [6.0, 15.6]	4.43 (87, 5)	<0.001	<0.001	0.92 [0.48, 1.35]
Midfoot	Right	56.2 ± 10.9	66.7 ± 12.2	10.5 [5.7, 15.3]	4.31 (86.9)	<0.001	<0.001	0.89 [0.46, 1.33]
Medial Heel	Left	73.4 ± 11.6	86.4 ± 16.2	13.0 [7.2, 18.8]	4.43 (87.0)	<0.001	<0.001	0.90 [0.47, 1.33]
Medial Heel	Right	72.2 ± 12.2	92.2 ± 14.6	20.0 [14.4, 25.6]	7.8 (87.8)	<0.001	<0.001	1.46 [0.99, 1.92]
Lateral Heel	Left	69.0 ± 13.2	74.0 ± 14.6	5.0 [−0.8, 10.8]	1.70 (86.6)	0.092	0.120	0.35 [−0.06, 0.77]
Lateral Heel	Right	69.6 ± 13.6	74.4 ± 14.4	4.8 [−1.1, 10.7]	1.62 (85.6)	0.109	0.139	0.34 [−0.08, 0.75]
Contact area (cm^2^)								
Hallux	Left	17.4 ± 2.6	20.5 ± 2.7	3.1 [2.0, 4.2]	5.53 (85.0)	<0.001	<0.001	1.16 [0.71, 1.60]
Hallux	Right	17.0 ± 2.4	21.4 ± 3.4	4.4 [3.2, 5.6]	7.18 (86.8)	<0.001	<0.001	1.45 [0.99, 1.92]
Toes 2–5	Left	12.3 ± 1.5	15.1 ± 2.3	2.8 [2.0, 3.6]	6.96 (84.8)	<0.001	<0.001	1.40 [0.94, 1.86]
Toes 2–5	Right	12.6 ± 1.4	14.5 ± 2.4	1.9 [1.1, 2.7]	4.69 (81.1)	<0.001	<0.001	0.93 [0.50, 1.37]
Metatarsal 1	Left	10.5 ± 1.8	12.9 ± 2.0	2.4 [1.6, 3.2]	5.98 (86.7)	<0.001	<0.001	1.24 [0.79, 1.69]
Metatarsal 1	Right	12.8 ± 1.3	18.5 ± 3.1	5.7 [4.7, 6.7]	11.77 (68.7)	<0.001	<0.001	2.29 [1.76, 2.82]
Metatarsal 2	Left	10.3 ± 1.2	12.4 ± 1.3	2.1 [1.6, 2.6]	7.95 (86.2)	<0.001	<0.001	1.66 [1.18, 2.14]
Metatarsal 2	Right	12.2 ± 1.3	14.7 ± 1.3	2.5 [2.0, 3.0]	9.07 (83.7)	<0.001	<0.001	1.91 [1.41, 2.41]
Metatarsal 3	Left	8.5 ± 1.0	10.2 ± 1.0	1.7 [1.3, 2.1]	8.01 (83.7)	<0.001	<0.001	1.69 [1.20, 2.17]
Metatarsal 3	Right	9.8 ± 1.3	11.6 ± 0.9	1.8 [1.3, 2.3]	7.45 (66.8)	<0.001	<0.001	1.63 [1.15, 2.11]
Metatarsal 4	Left	9.0 ± 1.1	10.6 ± 1.2	1.6 [1.1, 2.1]	6.58 (86, 3)	<0.001	<0.001	1.37 [0.91, 1.83]
Metatarsal 4	Right	9.7 ± 1.0	11.8 ± 1.1	2.1 [1.7, 2.5]	9.47 (86.5)	<0.001	<0.001	1.97 [1.47, 2.47]
Metatarsal 5	Left	11.7 ± 1.8	14.9 ± 1.9	3.2 [2.4, 4.0]	8.18 (85.5)	<0.001	<0.001	1.71 [1.23, 2.19]
Metatarsal5	Right	10.1 ± 1.7	11.9 ± 2.0	1.8 [1.0, 2.6]	4.61 (87.7)	<0.001	<0.001	0.95 [0.52, 1.39]
Midfoot	Left	35.5 ± 4.7	46.2 ± 7.8	10.7 [8.1, 13.3]	8.04 (82.3)	<0.001	<0.001	1.61 [1.13, 2.08]
Midfoot	Right	33.8 ± 4.5	40.8 ± 7.9	7.0 [4.4, 9.6]	5.28 (80.2)	<0.001	<0.001	1.05 [0.61, 1.49]
Medial Heel	Left	16.8 ± 1.9	20.7 ± 2.1	3.9 [3.1, 4.7]	9.23 (86.6)	<0.001	<0.001	1.92 [1.42, 2.42]
Medial Heel	Right	17.6 ± 2.1	21.3 ± 1.9	3.7 [2.8, 4.6]	8.66 (79.6)	<0.001	<0.001	1.84 [1.35, 2.34]
Lateral Heel	Left	14.2 ± 1.9	17.8 ± 1.8	3.6 [2.8, 4.4]	9.14 (81.6)	<0.001	<0.001	1.93 [1.43, 2.44]
Lateral Heel	Right	15.1 ± 1.8	18.2 ± 1.4	3.1 [2.4, 3.8]	8.94 (72.4)	<0.001	<0.001	1.93 [1.43, 2.43]

Values are reported as mean ± SD. Mean differences are calculated as male minus female. Sex comparisons were performed separately for the left and right feet using Welch’s independent-samples *t*-test. Two-sided *p*-values were adjusted for the 60 sex-related comparisons using the Benjamini–Hochberg false discovery rate procedure. Effect sizes are reported as Hedges’ g with 95% confidence intervals.

**Table 5:** Contextual comparison of the present plantar loading dataset with previously published data in healthy adults.

Study	Population	Measurement/ Protocol	Segmentation	Comparable Parameter(s)	Principal Published Findings	Relation to the Present Study
Xu et al. [20]	32 healthy adults; 15 F/17 M	Footscan^®^ platform; barefoot; 3 repeated trials	10 regions: hallux, T2–5, M1–M5, midfoot, medial heel, lateral heel	Maximum force; contact area	MaF was highest at medial heel: 460.7 ± 106.7 N, followed by M3: 370.1 ± 93.5 N, and lateral heel: 337.5 ± 82.4 N. CA was highest at midfoot: 38.4 ± 7.7 cm^2^, followed by medial heel: 21.8 ± 2.4 cm^2^ and lateral heel: 19.3 ± 2.1 cm^2^.	The regional pattern is broadly comparable. In the present sex- and side-specific dataset, corresponding mean values ranged from 280.2 to 360.9 N at medial heel, 174.1 to 238.5 N at M3, and 270.9 to 287.5 N at lateral heel. CA means ranged from 33.8 to 46.2 cm^2^ at midfoot, 16.8 to 21.3 cm^2^ at medial heel, and 14.2 to 18.2 cm^2^ at lateral heel
Koo et al. [26]	20 healthy young adults; 10 F/10 M; mean age 22.4 ± 1.2 y	Dynamic pedobarography during treadmill walking	5 regions: medial/lateral forefoot, medial/lateral midfoot, heel	Peak pressure; pressure-time integral	Men showed higher regional peak pressures, including medial forefoot (34.3 ± 6.3 vs. 25.9 ± 5.5 N/cm^2^, M vs. F).	Supports the presence of sex-related regional loading differences. Direct numerical comparison with the present study is not appropriate because the biomechanical outcomes and segmentation models differ
Yamamoto et al. [27]	100 healthy adults; 50 F/50 M	Thin in-shoe plantar pressure sensor; 200 Hz; ≥10 steps at comfortable speed; sex comparisons adjusted for BMI and gait speed	Sensor-based regional analysis including hallux and regional AP/ML divisions	Peak plantar pressure	Women showed significantly higher peak pressure at the hallux, toes, forefoot, and medial foot than men after adjustment.	The direction of several sex-related pressure differences differs from the absolute-force findings of the present study, emphasizing the influence of outcome definition, measurement system, walking conditions, and anthropometric adjustment.
Leyh & Feipel [28]	170 healthy adults; 83 F/87 M; 18–85 y	GAITRite^®^ 6 m instrumented pressure-sensitive walkway; slow, preferred, and fast self-selected walking speeds	6 regions: medial/lateral forefoot, midfoot, and heel	Relative peak pressure; PTI; contact area	Walking velocity significantly influenced several plantar pressure parameters, including peak pressure, PTI, and contact area, with region-dependent effects	Reinforces walking velocity as an important determinant of plantar loading and supports cautious interpretation of the present study, in which walking speed was not directly measured and total contact time was used only as a quality-control criterion.
Present study	90 healthy young adults	RSscan platform; barefoot; standardized single-step protocol; 3 valid recordings	10 regions: hallux, T2–5, M1–M5, midfoot, medial heel, lateral heel	Maximum force; impulse; contact area	Force-related loading was greatest predominantly in heel and central metatarsal regions, while contact area was greatest at the midfoot and heel. Multiple differences between female and male participants remained significant after FDR correction.	Provides a sex- and side-specific, platform- and population-specific descriptive reference dataset obtained under a standardized RSscan protocol.

## Data Availability

The original contributions presented in this study are included in the article. Further inquiries can be directed to the corresponding author.

## References

[1] Arellano-González J.C., Medellín-Castillo H.I., Cervantes-Sánchez J.J., Vidal-Lesso A. (2021). A Practical Review of the Biomechanical Parameters Commonly Used in the Assessment of Human Gait. Rev. Mex. Ing. Bioméd..

[2] Fukuchi C.A., Fukuchi R.K., Duarte M. (2019). Effects of Walking Speed on Gait Biomechanics in Healthy Participants: A Systematic Review and Meta-Analysis. Syst. Rev..

[3] Horst F., Lapuschkin S., Samek W., Müller K.-R., Schöllhorn W.I. (2019). Explaining the Unique Nature of Individual Gait Patterns with Deep Learning. Sci. Rep..

[4] Perry J., Burnfield J.M. (2010). Gait Analysis: Normal and Pathological Function.

[5] Sürer E., Köse A., Salah A.A., Gevers T. (2011). Methods and Technologies for Gait Analysis. Computer Analysis of Human Behavior.

[6] Brunnekreef J.J., van Uden C.J.T., van Moorsel S., Kooloos J.G.M. (2005). Reliability of Videotaped Observational Gait Analysis in Patients with Orthopedic Impairments. BMC Musculoskelet. Disord..

[7] Whittle M.W. (2007). Gait Analysis: An Introduction.

[8] Chiari L., Della Croce U., Leardini A., Cappozzo A. (2005). Human Movement Analysis Using Stereophotogrammetry: Part 2: Instrumental Errors. Gait Posture.

[9] Telfer S., Bigham J.J. (2019). The Influence of Population Characteristics and Measurement System on Barefoot Plantar Pressures: A Systematic Review and Meta-Regression Analysis. Gait Posture.

[10] Orlin M.N., McPoil T.G. (2000). Plantar Pressure Assessment. Phys. Ther..

[11] Pataky T.C., Goulermas J.Y. (2008). Pedobarographic Statistical Parametric Mapping (pSPM): A Pixel-Level Approach to Foot Pressure Image Analysis. J. Biomech..

[12] Buldt A.K., Murley G.S., Butterworth P., Levinger P., Menz H.B., Landorf K.B. (2018). The Relationship Between Foot Posture and Plantar Pressure During Walking in Adults: A Systematic Review. Gait Posture.

[13] Putti A.B., Arnold G.P., Cochrane L.A., Abboud R.J. (2007). The Pedar^®^ In-Shoe System: Repeatability and Normal Pressure Values. Gait Posture.

[14] Chockalingam N., Giacomozzi C., Healy A., Sacco I.C.N. (2025). Discrepancies between Plantar Pressure Devices: Evaluating Cross-System Reliability for Biomechanics, Clinical Use and Predictive Modelling. Foot.

[15] Van Der Velde G., Laloyaux H., Ronsse R. (2025). Inducing Asymmetric Gait in Healthy Walkers: A Review. Front. Rehabil. Sci..

[16] Cartón-Llorente A., Cardiel-Sánchez S., Molina-Molina A., Ráfales-Perucha A., Rubio-Peirotén A. (2024). Bilateral Asymmetry of Spatiotemporal Running Gait Parameters in U14 Athletes at Different Speeds. Sports.

[17] Kadaba M.P., Ramakrishnan H.K., Wootten M.E. (1990). Measurement of Lower Extremity Kinematics During Level Walking. J. Orthop. Res..

[18] Sutherland D.H. (1997). The Development of Mature Gait. Gait Posture.

[19] Hollman J.H., McDade E.M., Petersen R.C. (2011). Normative Spatiotemporal Gait Parameters in Older Adults. Gait Posture.

[20] Xu C., Wen X.X., Huang L.Y., Shang P., Ma S., Zhao Y., Fan Y., Zhang M. (2017). Normal Foot Loading Parameters and Repeatability of the Footscan Platform System. J. Foot Ankle Res..

[21] Arzehgar A., Nia R.G.N.N., Hoseinkhani M., Masoumi F., Sayyed-Hosseinian S.H., Eslami S. (2025). An Overview of Plantar Pressure Distribution Measurements and Its Applications in Health and Medicine. Gait Posture.

[22] Burnie L., Chockalingam N., Holder A., Claypole T., Kilduff L., Bezodis N. (2024). Testing Protocols and Measurement Techniques When Using Pressure Sensors for Sport and Health Applications: A Comparative Review. Foot.

[23] Detels K., Shin D., Wilson H., Zhou S., Chen A., Rosendorf J., Taseh A., Akhbari B., Schwab J.H., Ghaednia H. (2024). Clinical Applications of Plantar Pressure Measurement. arXiv.

[24] Razak A.H.A., Zayegh A., Begg R.K., Wahab Y. (2012). Foot Plantar Pressure Measurement System: A Review. Sensors.

[25] Ramanathan A.K., Kiran P., Arnold G.P., Wang W., Abboud R.J. (2010). Repeatability of the Pedar-X^®^ In-Shoe Pressure Measuring System. Foot Ankle Surg..

[26] Koo S., Chun S., Lee K.M., Cho B.C., Koo Y.J., Kang D.W., Park M.S. (2018). Sex Differences in Pedobarographic Findings and Relationship between Radiographic and Pedobarographic Measurements in Young Healthy Adults. Clin. Orthop. Surg..

[27] Yamamoto T., Hoshino Y., Kanzaki N., Nukuto K., Yamashita T., Ibaraki K., Nagamune K., Nagai K., Araki D., Matsushita T. (2020). Plantar Pressure Sensors Indicate Women to Have a Significantly Higher Peak Pressure on the Hallux, Toes, Forefoot, and Medial of the Foot Compared to Men. J. Foot Ankle Res..

[28] Leyh C., Feipel V. (2022). Impact of Sex and Velocity on Plantar Pressure Distribution during Gait: A Cross-Sectional Study Using an Instrumented Pressure-Sensitive Walkway. J. Funct. Morphol. Kinesiol..

[29] Chung M.J., Wang M.J.J. (2010). The Change of Gait Parameters during Walking at Different Percentages of Preferred Walking Speed for Healthy Adults Aged 20–60 Years. Gait Posture.

[30] Gurney J.K., Kersting U.G., Rosenbaum D. (2008). Between-Day Reliability of Repeated Plantar Pressure Distribution Measurements in a Normal Population. Gait Posture.

[31] Latour E., Latour E.E., Arlet J. (2024). Regional Differences in the Biological Variability of Plantar Pressure as a Basis for Refining Diagnostic Gait Analysis. Sci. Rep..

[32] Riskowski J.L., Hagedorn T.J., Dufour A.B., Hannan M.T. (2012). Functional Foot Symmetry and Its Relation to Lower Extremity Physical Performance in Older Adults: The Framingham Foot Study. J. Biomech..

[33] Patterson K.K., Gage W.H., Brooks D., Black S.E., McIlroy W.E. (2012). Temporal Gait Symmetry and Velocity Differ in Their Relationship to Age in Healthy Adults. Gait Posture.

[34] Kowalski E., Catelli D.S., Lamontagne M. (2019). Side Does Not Matter in Healthy Young and Older Individuals—Examining the Importance of How We Match Limbs during Gait Studies. Gait Posture.

[35] Matei D., Traistaru R., Amzolini A.M., Ianosi L.S., Neagoe C.D., Mitrea A., Clenciu D., Avramescu T.E. (2024). A Comparative Study on the Pain Threshold Experienced by Fibromyalgia Patients Following Acute SARS-CoV-2 Infection. Life.

[36] Amzolini A.M., Neagoe C.D., Avramescu T.E., Mitrea A., Traistaru R., Micu E.S., Ianoşi S.L., Matei D. (2024). Understanding non-pharmacological treatments for fibromyalgia functional and well-being status: The role of literacy. Healthcare.

